# Multiple Trichoepitheliomas: Cosmetic Improvement with Dermabrasion

**DOI:** 10.4103/0974-2077.79203

**Published:** 2011

**Authors:** Chekuri Raghuveer, Chidambara Murthy, Karijigi Siddalingappa, Doddarangaiah R Shivanand

**Affiliations:** *Department of Dermatology and Venereology, Vijayanagara Institute of Medical Sciences (VIMS), Bellary, India*; 1 *Department of Dermatology and Venereology, Siddartha Medical College, Tumkur, Karnataka, India. E-mail: c_raghuveer@yahoo.com*

Sir,

Trichoepitheliomas are well-differentiated benign follicular tumours. Clinically, trichoepitheliomas may present as solitary, multiple or desmoplastic lesions.[1] Patients with multiple trichoepitheliomas (MT) usually present to the clinician due to their location over the face and the large number of lesions. We report a case of MT, with a good therapeutic response to dermabrasion.

**Figure 1:**
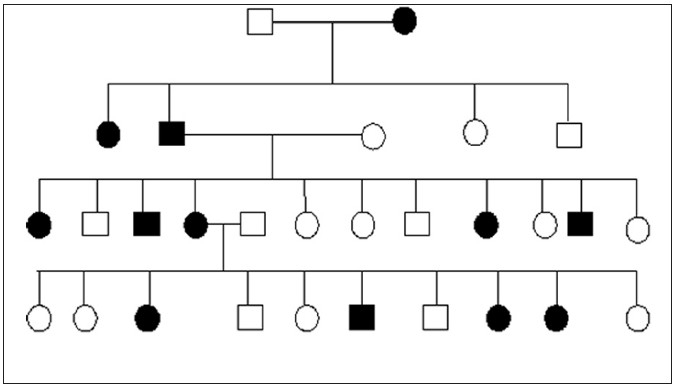
Pedigree chart

**Figure 2:**
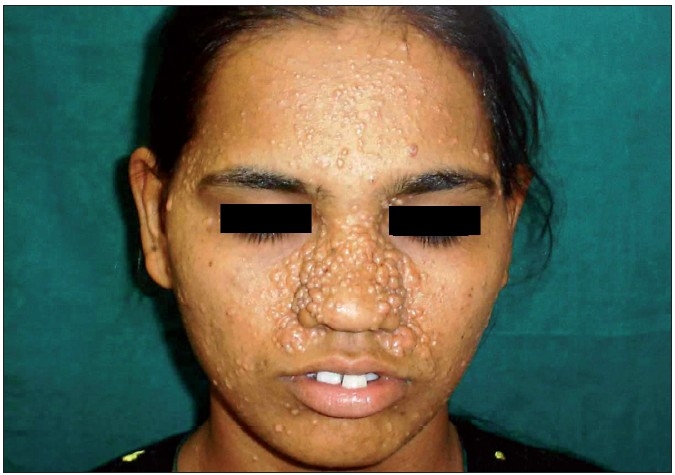
Multiple trichoepitheliomas over face

A 29-year-old woman presented with multiple, asymptomatic lesions over the face for the last 15 years. Onset was insidious with gradual increase in size and number. There was no history of drug intake or systemic complaints. Family history of similar involvement was present [Figure 1]. Significant spontaneous regression/disappearance of lesions over her mother’s face was noted during the last few years. Cutaneous examination showed bilateral multiple extensive non-tender round to oval skin-coloured papules and nodules measuring about two mm to one cm, over the face [Figure 2]. Involvement was less severe in her mother and younger sister. Other mucocutaneous, appendageal and systemic examinations were normal.

**Figure 3:**
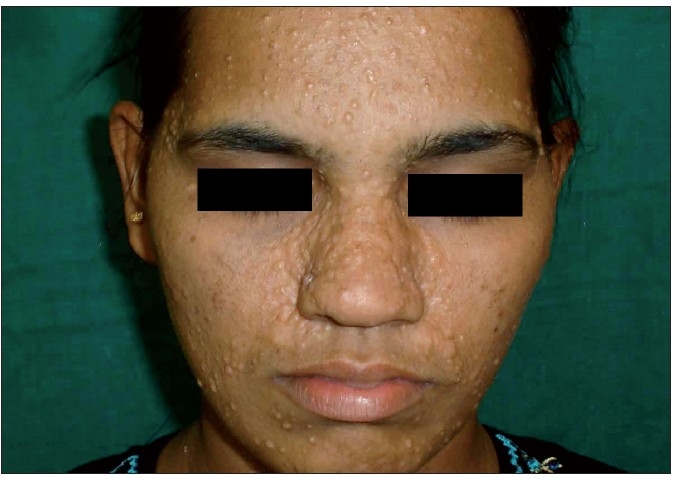
Improvement after dermabrasion

Her routine haematological, urine and biochemical investigations were normal. Blood VDRL, HIV were negative. Biopsy from one of the lesions showed features consistent with trichoepitheliomas. Patient was counselled to undergo dermabrasion. After informed consent, full face dermabrasion using a diamond-tip motor dermabrader under general anaesthesia with tumescent anaesthesia was done upto the junction of the upper and mid-reticular dermis. Collagen dressings, antibiotics, and anti-inflammatory drugs were given for a week following the procedure. She was also advised sun-protection measures. Significant cosmetic improvement without appearance of new lesions was noted during a one-year follow-up period [Figure 3].

MT is familial and inherited as autosomal dominant condition. Both sexes may be affected, although females outnumber males.[2] The gene for MT is located on Chromosome 9p21.[3] The tumours appear at puberty, are symmetrical, small, rounded and shiny. Commonly, cheeks, nasolabial folds and forehead are involved. Rare sites of involvement include neck, chest, shoulders and interscapular regions. The lesion may vary from a papule to a large nodule or tumour. Rarely, ulceration may occur simulating basal cell carcinoma.[2]

MT may be seen in Brooke-Spiegler syndrome, Rombo syndrome and Bazex syndrome.[3] Our patients did not have features suggestive of these disorders. Histology shows typical horn cysts, tumour islands composed of basophilic cells arranged in a network, foreign body giant reaction around horn cysts and differentiation towards hair structures.[2]

Onset at puberty, spontaneous regression of tumours postmenopausally in the mother, as in our case and female preponderance, may suggest a possible hormonal influence. Sawchunk and Herald[4] found no oestrogen and progesterone receptors on tumour tissue in a study of a single patient. Presently, we could not conduct studies in this regard, due to the lack of facilities.

Patients with MT usually seek treatment for cosmetic appearance. They also need to be followed up regularly due to the risk of developing basal cell carcinoma. Treatment of MT is disappointing and difficult. The lesions are situated in the deep dermis and liable for regrowth if partially removed. New lesions may continue to occur in MT, as it is genetically inherited.[5] Treatment modalities suggested include excision, dermabrasion, electrodessication, cryotherapy, radiotherapy, and Argon, CO2 and erbium-YAG lasers.[3]

Although, lasers have been found to give better results, they are not easily available, are expensive and may result in pigmentary problems in Indian skin.[5] Dermabrasion is equally effective, cheap, gives good and long-term cosmetic improvement, as in our patient. In conclusion, we report a large family pedigree with MT. Spontaneous regression of tumours in the mother is a rare and unusual finding. Dermabrasion, as in our case, is a good therapeutic option for MT in a resource-poor setting where lasers are not easily available or when not affordable by the patients. Further studies on oestrogen and progesterone receptors of tumour tissue in MT are suggested, as it may have a therapeutic implication with hormonal preparations.
